# Dynamics of *ex vivo* cytokine transcription during experimental *Toxocara canis* infection in Balb/c mice

**DOI:** 10.1590/S1984-29612024017

**Published:** 2024-03-18

**Authors:** Neida Lucia Conrad, Vitória Sequeira Gonçalves Zorzi, Natália Berne Pinheiro, Jéssica Lopes Borchard, Micaele Quintana de Moura, Fábio Pereira Leivas Leite

**Affiliations:** 1 Programa de Pós-graduação em Biotecnologia, Centro de Desenvolvimento Tecnológico, Universidade Federal de Pelotas - UFPel, Pelotas, RS, Brasil; 2 Programa de Pós-graduação em Microbiologia e Parasitologia, Instituto de Biologia, Universidade Federal de Pelotas - UFPel, Pelotas, RS, Brasil

**Keywords:** Toxocariasis, cell immune response, nematode parasite, Toxocaríase, resposta imune celular, parasita nematódeo

## Abstract

The cytokine microenvironment is crucial in generating and polarizing the immune response. A means of monitoring this environment would be of great value for better understanding *Toxocara canis* immune modulation. The aim of this study was to analyze the dynamics of cytokine transcription *ex vivo*, during early (24-48 hours) and late (15-30 days) times post-infection, in the mesenteric lymph nodes, spleen and intestinal mucosa of Balb/c mice experimentally infected with *T. canis* larvae. Mice in the treated group were infected with 100 third-stage larvae (L3), whereas mice in the control group were not infected. Analyses were performed at different times: 24-48 hours post-infection (HPI), 15-30 days post-infection (DPI). *IL4, IL10, IL12* and *Ym1* mRNA transcriptions were analyzed through qPCR. This study showed cytokine transcription mediated by migrating larvae in the mesenteric lymph nodes and spleen at 24-48 HPI, whereas cytokine transcription in the intestinal mucosa was observed only at late times (15-30 DPI). These results suggest that the *T. canis* larvae migration during infection might play a role in cytokine dynamics. Since the cytokine microenvironment is crucial in modulating immune response, knowledge of cytokine dynamics during *T. canis* infections pave the way to better understand its interaction with the host.

## Resumo

O microambiente das citocinas é crucial na geração e polarização da resposta imune. Monitorar esse ambiente é importante para entender a modulação imune de *Toxocara canis*. Este estudo analisou a dinâmica da transcrição de citocinas *ex vivo*, durante os períodos pós-infecção precoce (24-48 horas) e tardio (15-30 dias) em linfonodos mesentéricos, baço e mucosa intestinal em camundongos, infectados experimentalmente com larvas de *T. canis*. Camundongos foram infectados com 100 larvas de terceiro estágio (L3), enquanto os camundongos do grupo controle não foram infectados. Foram analisados diferentes períodos: 24-48 horas pós-infecção (HPI), 15-30 dias pós-infecção (DPI). A transcrição do mRNA de *IL4, IL10*, *IL12* e *Ym1* foi analisada por qPCR. Observou-se a transcrição de citocinas mediada por larvas migratórias nos linfonodos mesentéricos e baço por 24-48 HPI, enquanto na mucosa intestinal a transcrição de citocinas foi observada apenas na infecção tardia (15-30 DPI). Esses resultados sugerem que a migração das larvas de *T. canis*, durante a infecção, pode desempenhar um papel na dinâmica das citocinas. Uma vez que o microambiente das citocinas é crucial na modulação da resposta imune, o conhecimento da dinâmica das citocinas, durante as infecções por *T. canis*, permite uma melhor compreensão da sua interação com o hospedeiro.

## Introduction

*Toxocara canis* is a parasitic intestinal nematode with worldwide distribution that can infect humans (Ma et al., 2018). Human toxocariasis is prevalent across the globe and has been considered to be the most important neglected zoonotic parasitic infection (Ma et al., 2020). After somatic migration, *T. canis* larvae do not develop in the paratenic host. Instead, they can evade the host immune response by migrating through different tissues and, in so doing, can cause a broad range of clinical symptoms with high importance in relation to for human health (Ma et al., 2018).

*Toxocara canis* is able to modulate the innate and adaptive immune responses of its definitive and paratenic hosts. The immune modulatory mechanism of *T. canis* infection is not totally understood, but it is well known that adaptive immunity to *Toxocara* is predominantly characterized by CD4+ T helper cells converting to type 2 subsets (Th2), with the release of type 2 cytokines (e.g. IL-4 and IL-10) (Maizels, 2013). *Toxocara canis* invasion leads to suppression of the Th1 response through downregulating IL-12 and IFN-y (Avila et al., 2016) and promoting stimulation of alternatively activated macrophages (AAMacs), such as M2, towards a Th2 immune response (Edwards et al., 2006; Gordon, 2003).

Cytokines play an important role in modulating the type of immune response (e.g. Th-1, Th-2, Th-17 or Treg) (Kaufmann, 2007). The presence of IL-4 in the early stages of T cell priming induces an immune response toward Th-2 (Noben-Trauth et al., 2000, 2002). Parasites can evade the host immune system through activation of regulatory T cells such as IL-10 (Gasbarre, 1997; Maizels, 2013). Production of IL-10 by macrophages and dendritic cells (DCs) plays a role in directing a polarizing Th2-type response through suppressing the Th1-type response (Oleszycka et al., 2018).

Cytokines that belong to the IL-12 family are essential for the development of innate and acquired immunity (Ma et al., 2015; Xi & Jin, 1998). *Toxocara canis* is able to suppress IL-12 expression and, by doing so, hinders the recruitment and activation of immune cells, thereby favoring infection (Kuroda et al., 2001).

Mechanisms that are either dependent on or independent of IL-4 can induce activation of M2 in parasite infections, and play a role in enhancement of Th2 cell differentiation and suppression of Th1 responses (Raes et al., 2002; Welch et al., 2002). The protein Ym1 is considered to be a marker of AAMacs (Faz-López et al., 2019) and is the most abundantly expressed gene in helminth parasite-activated macrophages (Welch et al., 2002).

Understanding the immunological changes that occur in the experimental *T. canis* murine model is extremely important with regard to increase knowledge about toxocariasis. Since the cytokine microenvironment is crucial in generating and polarizing the immune response, a means of monitoring this environment would be of great value for better understanding *T. canis* immune modulation.

Thus, here we analyzed the dynamics of cytokine transcription *ex vivo*, at early (24-48 hours) and late (15-30 days) times post-infection, in the mesenteric lymph nodes, spleen and intestinal mucosa of Balb/c mice experimentally infected with *T. canis* larvae.

## Material and Methods

### Mice

A total of 80 female Balb/c mice (5-6 weeks old) were randomly separated into five groups, of which four were experimental and contained 10 animals each, and one was the control group containing a total of 40 mice. The animals were kept in a controlled environment (22 ± 2 °C) that was subjected to 12-h light/dark cycles, with *ad libitum* access to food and water.

### Obtaining *T. canis* larvae

*T. canis* eggs were collected from a naturally infected donor dog, and were incubated as described by Avila et al. (2012). Larvae were extracted from the eggs by physicochemical methods, as described by De Savigny (1975) and were maintained in culture as proposed by Falcone et al. (2001) and Perkins (1992). The viability of the larvae was confirmed by examining their motility under a microscope.

### Experimental design

Experimental infection was performed by means of oral administration of 100 larvae (L3) of *T. canis* per mouse (Moura et al., 2020), using an intragastric tube (Avila et al., 2012). The mice of groups 1 to 4 (n = 10 each) were infected with 100 infective larvae (L3), whereas the mice of group 5 (control, n = 40) were not infected. The experimental groups were euthanized at different times: Group 1, 24 hours post-infection (HPI); Group 2, 48 HPI; Group 3, 15 days post-infection (DPI); and Group 4, 30 DPI. At each time point studied, 10 mice from the control group were also euthanized. To confirm *T. canis* larval infection, recovery of larvae was performed at 24 and 48 HPI using the liver tissue digestion technique (Xi & Jin, 1998).

### Tissue preparation

Tissue preparation was done as described by Moura et al. (2018). Briefly, the duodenum mucosa was gently scraped to collect cells. The material recovered consisted mainly of enterocytes, with scattered goblet cells and occasional enteroendocrine cells. In crypts, the material also included Paneth cells and stem cells. After collection, the cells were washed twice in sterile Hank's solution. TRIzol® (Sigma-Aldrich) was then added and the material was then stored at −70 °C.

Mesenteric lymphoid nodules located between the leaflets of the mesentery, from the duodenum to the rectum, were also collected. These were washed twice in sterile Hank’s solution (without Ca and Mg) and macerated. Cells were then collected and suspended in sterile Hank's solution, after which the cell pellets were suspended in TRIzol (Sigma-Aldrich) and stored at −70 °C.

The spleens were removed and macerated, and the splenocytes thus obtained were suspended in a balanced Hank’s solution. Afterward, the cells were centrifuged, and the pellet was suspended in lysis solutions (0.8% ammonium chloride), followed by washing and suspension in sterile Hank's solution (Cultilab, Campinas, Brazil). TRIzol was then added and the material was stored at −70 °C.

### qPCR analysis of *lL4, IL10, IL12* and *Ym1* mRNA transcription

RNA extraction was performed using the TRIzol method and cDNA synthesis from 400 ng/ μl of mRNA, in accordance with the manufacturer’s instructions (Applied Biosystems, Foster City, CA, USA). For each time point studied, a pool of tissue from ten animals were evaluated in duplicate. The quantitative polymerase chain reaction method (qPCR) was used, with the following primers for amplification of gene segments: *IL4* gene (F: CCAAGGTGCTTCGCATATTT, R: ATCGAAGAGTAGGAGT, R: TTTGTCCTTAGGAGGGCTTCCTCG); *IL10* gene (F: TTTGAATTCCCTGGGTGAGAA, R: ACAGGGGAGAAATCGATGACA); *IL12* gene (F: AGCACCAGCTTCTTCATCAGG, R: CCTTTCTGGTTACACCCCTCC); *Ym1* gene (F: CACAGGTCTGGCAATTCTTCTG, R: TTTGTCCTTAGGAGGGCTTCCTCG); and *β actin* (F: AACGCCCTTCATTGAC, R: TCCACGACATACTCAGCAC). The qPCR runs were performed with 1 μL of cDNA, 5.0 μL of SYBR Green (Invitrogen, Carlsbad, CA, USA), 0.25 μM of each primer oligomer and 3.5 μL of RNase-free water (Gibco, Gaithersburg, MD, USA), in a total volume of 10 μL. The temperatures used were as follows: denaturation at 95 °C for 5 min, followed by 40 cycles with denaturation at 95 °C for 30 s, annealing at 65 °C for 60 s and extension at 72 °C for 60 s, and final extension at 72 °C for 5 min.

All samples were analyzed in triplicate. From the cycle threshold (Ct) values obtained, the variation in gene transcription was calculated through comparison with the expression of *β actin* (control). The 2^-(ΔΔCt)^ method was used for relative quantification of gene transcription between samples (Livak & Schmittgen, 2001).

### Statistical analysis

Expression levels of all target genes were calculated relative to the housekeeping gene *β-actin*. Two-way ANOVA followed by Dunnett's multiple-comparison test was used to analyze relative gene transcription. All statistical analyses were performed in GraphPad Prism version 9.1.1 for macOS (GraphPad Software, USA) and a *p*-value of ≤ 0.05 was considered statistically significant.

## Results

### Infection

*Toxocara canis* infection was confirmed in terms of the mean larval recovery at 24 and 48 HPI from all infected mice livers. The mean recovery observed was 9.5 ± 3.4 larvae at 24 HPI and 13.3 ± 7.2 at 48 HPI.

### Cytokine transcription

**Figure 1:**
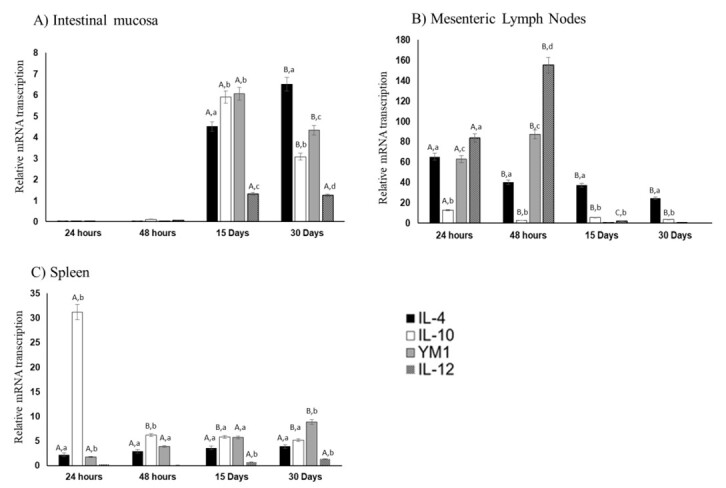
Relative mRNA transcription of the cytokines *IL4*, *IL10*, *IL12* and *YM1* in intestinal mucosa (A), mesenteric lymph nodes (B) and spleen (C) of mice experimentally infected with 100 viable *T. canis* larvae, at 24 and 48 hours post-infection, and at 15 and 30 days post-infection. The data comprise mean +/- standard deviation relating to transcription of genes from three pools of tissue collected from 10 mice per group in duplicate, calculated using threshold cycle (Ct) values in the 2^-ΔΔCt^ formula. A non-infected group was used as a control parameter. Different lowercase letters represent significant differences seen through Dunnett's multiple-comparison test between different cytokines at the same time, while different uppercase letters represent differences in the same cytokine between the times evaluated.

The transcription patterns were different among some cytokines studied, differing both between the early (24-48 HPI) and late (15-30 DPI) evaluation time. Regarding the mucosal tissue, we did not observe any transcription of the genes studied, compared with the control mice, at 24-48 HPI (Figure 1). However, at 15 and 30 DPI, significant levels of transcription of *IL4, IL10, IL12* and *YM1* occurred. The *IL4* transcription was ~4.5-fold higher at 15 DPI and reached ~6.5-fold higher at 30 DPI, compared with the control group (Figure 1A). It was noted that *IL4* was the only cytokine for which there was a significant increase (p < 0.05) in transcription expression from 15 to 30 DPI. For *IL10* and *Ym1*, the transcription expression significantly decreased (p < 0.05) between 15 and 30 DPI, whereas the expression of *IL12* remained steady over this period of time (Figure 1A).

The cytokine transcription in the mesenteric lymph nodes showed significant expression of all cytokines at the early infection stage (Figure 1B). It was noteworthy that there was a significant reduction in transcription (p < 0.05) for *IL4* and *IL10* from 24 to 48 HPI, whereas for *YM1* and *IL12* there was a significant increase (p < 0.05) from 24 to 48 HPI. The transcription of *IL4* and *IL10* was significant at 15 and 30 DPI, however it was lower (p < 0.05) than at the initial stages (24-48 HPI) (Figure 1B).

The cytokine transcription results from the splenocytes showed that there was significant transcription expression for *IL4*, *IL10* and *YM1*, but not for *IL12*, in comparison with the controls. Surprisingly, the expression of *IL10* at 24 HPI was significantly greater (p < 0.05) than that of the other cytokines (Figure 1C). The transcription levels observed at the late stage of infection were similar for *IL4*, *IL10* and *IL12* but there were significantly increases for *YM1* from day 15 to day 30 DPI (Figure 1C).

## Discussion

Better understanding of toxocariasis in humans has been achieved through studies using mouse experimental models (Moura et al., 2018). These have revealed key aspects of the immune modulation mediated by *T. canis* (Maizels, 2013). Cytokines are mediators that regulate immune responses and, thus, knowledge of cytokine dynamics during parasite infections can provide valuable information on the immune interactions between host and pathogen.

It is well known that *T. canis* larvae invade from the host’s intestinal tract and disseminate throughout the tissues of the body. Concerning the migratory route of *T. canis* larvae, a previous review by Othman showed that there have been controversies about the migratory route and accumulation of larvae in different organs during the acute and chronic phases. It can be speculated that the order of organ infection during an active larvae migration might play a role in the immune modulation mediated by *T. canis* (Othman et al., 2011). With the aim of studying the immune modulation of early (24-48 HPI) and late (15-30 DPI) *T. canis* infection in mice, we evaluated the dynamics of cytokine transcription in the mesenteric lymph nodes, spleen and intestinal mucosa. The design of the present study was based on periods and points described by Borchard et al. (2022).

We were able to clearly demonstrate that cytokine transcription mediated by migrating larvae in the mesenteric lymph nodes and spleen was already occurring by 24-48 HPI, whereas in the intestinal mucosa, cytokine transcription was observed only at the late stage of infection (15-30 DPI). These results suggest that there is a *T. canis* larval migration route in BALB/c mice and that cytokine transcription dynamics follow an infection pathway.

According to the literature we can mention some aspects such as during the course of the immune response to *T. canis* infection, there is a predominance of a Th2 immune response, characterized by the production of IL4, IL5, IL13 and IgG1 and IgE immunoglobulin subclasses, as well as an increase in eosinophils in peripheral blood and eosinophils in the lung and liver (Pinelli et al., 2007). On the other hand, the production of Th1 lymphocytes is suppressed during *T. canis* infection and the production of IL12 and TNF-α, important cytokines involved in cellular recruitment, are reduced in macrophages from infected animals. The suppression of Th1 lymphocytes, associated with the reduction of IL12, is an immunomodulatory characteristic of the nematode *T. canis*, which hinders the recruitment of macrophages to sites of infection and parasite death (Kuroda et al., 2001).

A widely accepted hypothesis for the function of mediators involved in the modified Th2 response against helminths is that helminth PAMPs can stimulate Th1 cell-biased dendritic cells into Th2 or regulatory T cell (Treg) subsets. Treg lymphocytes, induced in parallel, produce suppressive cytokines such as IL10 and TGF-β and mitigate the levels of innate and adaptive immune activation (Allen & Sutherland, 2014). Ym1 protein is expressed gene in helminth-activated macrophages (Loke et al., 2002) and may be activated by helminth infection independently of IL4/IL13 signaling. Studies reported that experimental mice infected with *T. canis* larvae have an upregulation of Ym1, which favors parasite resistance (Faz-López et al., 2019).

We observed increases in *IL4* and *IL10* transcription at early infection times (24-48 HPI) in the mesenteric lymph nodes. It is well known that *T. canis* infection induces a polarized Th2 response, typically reflected by the presence of IL-4 and by activation of regulatory T cells, which bring production of downregulating cytokines, such as IL-10 (Fialho et al., 2018; Pinelli et al., 2005). Our finding is similar to what was reported previously regarding *IL4* and *IL10* transcription in Balb/c mice lymph nodes during early *T. canis* infection (72h) (Tawill et al., 2004). Tissue damage caused by migration of helminths triggers stress responses associated with type 2 inflammation. This activates a variety of cell types of the innate immune system, including basophils, mast cells, natural killer T cells, innate lymphoid cells (ILCs), neutrophils and M2 macrophages. M2 macrophages are the key for promoting Th2 responses and suppressing Th1-driven inflammatory pathology, and YM1 is a marker for this stimulation. It should be noted that the evolution of *YM1* transcription over the period from 24 to 48 HPI followed the same *IL4* upregulation pattern (Figures 1A, B and C), thus suggesting that M2 macrophage activation was mediated by IL4 (Donnelly et al., 2008).

The initial signal triggering IL-12 expression is the exposure to microorganisms (e.g. parasites) at lymph nodes (Heufler et al., 1996; Hsieh et al., 1993). In the present study, we observed significant *IL12* transcription at 24 HPI, which became greater by 48 HPI. It should be noted that significant *IL12* transcription was observed only at lymph nodes and during early infection, which thus illustrates the complexity of the immune modulation mediated by *T. canis* at the early stage of infection (Figures 1A, B and C).

Upon evaluating *IL4* and *IL10* transcription in mesenteric lymph nodes at late time points (15-30 DPI), we still observed significant transcription of *IL4* and *IL10*, but with a significant reduction. For *YM1*, we observed the same tendency, with less transcription, following the reduction in *IL4* transcription. These findings support the biological relevance of *IL4* in *YM1* expression and corroborate the observations reported by Welch et al. (2002).

The spleen has important immunological functions with regard to protecting the host against pathogens (Lewis et al., 2019), but the role of the spleen in *T. canis* infection is not totally understood. At 24-48 HPI, we observed significant upregulation of *IL4*, *IL10* and *YM1*, but no *IL12* transcription. This finding is quite interesting, since significant *IL12* transcription was observed at lymph nodes at early infection times (24 and 48 HPI). This suggests that the organ involved and the time of *T. canis* infection play a role in the immune modulation mechanism, since at late stages of infection (15-30 DPI), increased *IL12* transcription was observed.

In evaluating the intestinal mucosa, no cytokine or *YM1* transcription was observed at 24-48 HPI (Figures 1A, B and C). These observations are in line with the immune response observed in the mucosa of infected mice at early infection times (5-9 HPI) reported by Abo-Shehada et al. (1991). However, at late infection times (15-30 DPI), significant transcription upregulation was observed for all the genes studied. It is well known that immune cells circulate through the mesenteric lymph nodes, to enter the blood via the thoracic duct and return to the intestinal mucosa, thereby repopulating distant mucosal sites (Iwasaki & Medzhitov, 2010). So, it can be suggested that if such events occur during *T. canis* infection, this may reflect the findings observed at 15-30 DPI in the intestinal mucosa. This therefore suggests that larvae migration through mesenteric lymph nodes is the starting point for immune modulation mediated by cytokines during *T. canis* infection.

Our study had some limitations. Firstly, we did not identify the cell population expressing the cytokines evaluated, or YM1, in each tissue studied. This is very important, since identifying the cell population that is stimulated by *T. canis* larvae and modulates gene transcription is a key point for better understanding the immune modulatory mechanism of *T. canis*. Secondly, we did not identify which *T. canis* virulent factor (e.g. TES) was involved in the immune modulation. However, the data obtained in the present study may help towards better understanding of some of the immune modulatory mechanism mediated by *T. canis* infection.

According to the study developed it is believed that immunological modulation begins when the larvae reach the mesenteric lymph nodes (24-48 HPI), a fact evidenced by the significantly higher transcription of cytokines in this organ, compared to the others. Subsequently (15-30 DPI) there is a significant reduction in the transcription of cytokines in the lymph nodes, while transcription is accentuated in the intestinal mucosa, an organ that already has larval colonization established during this period. When evaluating the transcription of cytokines in the spleen, the presence of cytokines was observed in the early and late periods of infection.

## Conclusion

We presented new insights into cytokine dynamic transcription responses in experimentally infected BALB/c mice during the early and late stages of *T. canis* infection. The results will pave the way to better understanding of immune modulation events that occur during toxocariasis and, in so doing, may contribute to its control.
